# Cross-cultural adaptation of the Schizophrenia Caregiver Questionnaire (SCQ) and the Caregiver Global Impression (CaGI) Scales in 11 languages

**DOI:** 10.1186/s12955-015-0264-y

**Published:** 2015-06-09

**Authors:** Diana Rofail, Catherine Acquadro, Cécile Izquierdo, Antoine Regnault, Steven H. Zarit

**Affiliations:** Patient-Centered Outcomes Research for Neuroscience & Metabolism, Roche Products Ltd, Welwyn Garden City, UK; Mapi Research Trust, Lyon, France; Language Services, Mapi, Lyon, France; HEOR & Strategic Market Access, Mapi, 27 rue de la Villette, 69003 Lyon, France; Department Head, Human Development and Family Studies, The Pennsylvania State University, University Park, PA USA

**Keywords:** Schizophrenia, Caregiver, Clinical outcome assessment, Translation, Cross-cultural adaptation

## Abstract

**Background:**

The Schizophrenia Caregiver Questionnaire (SCQ) was developed to provide a comprehensive view of caregivers’ subjective experiences of the impacts of caring for someone with schizophrenia. The Caregiver Global Impression (CaGI) scales were designed to assess their perception of the severity of the schizophrenia symptoms, of change in schizophrenia symptoms and in the experience of caring since the beginning of the study. The objectives of the study were to translate the SCQ and CaGI scales in 11 languages [French (Canada, France), English (Canada, UK, Australia), German (Germany), Italian (Italy), Spanish (Spain), Dutch (the Netherlands), Finnish (Finland), and Swedish (Sweden)], to present evidence that the translations capture the concepts of the original questionnaires and are well understood by caregivers of patients with schizophrenia in each target country.

**Methods:**

The different language versions were developed using a standard or adjusted linguistic validation process fully complying with the International Society for Pharmacoeconomics and Outcomes Research (ISPOR) recommended procedures.

**Results:**

Interviews were conducted with 55 caregivers of patients with schizophrenia from 10 countries representing the 11 different languages. Participants ranged in age from 28 to 84 years and had 5 to 16 years of education. Women represented 69.1 % (38/55) of the sample. Fourteen out of the 32 items of the SCQ generated difficulties which were mostly of semantic origin (13 items). The translation of the CaGI scales did not raise any major difficulty. Only five out of the 55 caregivers had difficulty understanding the meaning of the translations of “degree” in the expressions “degree of change in experience of caring” and “degree of change in symptoms”.

**Conclusions:**

Translations of the SCQ and CaGI scales into 11 languages adequately captured the concepts in the original English versions of the questionnaires, thereby demonstrating the conceptual, semantic, and cultural equivalence of each translation.

## Background

Schizophrenia is a severe mental illness affecting 0.3-0.7 % of the population worldwide [1], characterized by three domains of psychopathology, including negative symptoms (social withdrawal, lack of motivation and emotional reactivity), positive symptoms (hallucinations, delusions) and cognitive deficits (working memory, attention executive function) [2]. It is considered a leading cause of disability [3].

With the transition in care from formal hospital based healthcare systems to outpatient and community services and informal caregivers, it is estimated that 50-90 % of people with chronic psychiatric illness live with their families or friends [4, 5]. Informal caregivers (defined as “*a person who has significant responsibility for managing the well-being of a person diagnosed with schizophrenia in an unpaid capacity”* [6]*)* provide an important service by reducing the need for formal care and the burden upon healthcare systems [7].

The Schizophrenia Caregiver Questionnaire (SCQ) was developed to provide a comprehensive view of caregivers’ subjective experiences of the impacts of caring for someone with schizophrenia [8]. It was adapted from the Zarit Burden Interview (ZBI) [9] in accordance with best practice recommendations for the development and modification of self-report measures [10]. The ZBI was originally developed to measure subjective burden among caregivers of adults with dementia. Items were generated based on clinical experience with caregivers and prior studies resulting in a 22-item self-report inventory that examines burden associated with functional/behavioural impairments and the home care situation. Based on findings from the literature, review of the ZBI, expert opinion, and face-to-face, semi-structured interviews with 19 US English speaking caregivers [11], changes were made to address concerns regarding relevance and sensitivity of the ZBI in the population of caregivers of patients with schizophrenia. The main changes consisted in the rewording of some items, the development of eleven additional items focusing on issues important to caregivers, the deletion of one item (i.e., item 9 of the ZBI - *Do you feel strained when you are around your relative?*), and the addition of a recall period (i.e., the past four weeks). In addition, the 5-point Likert-type response scale was replaced by an 11-point numerical rating scale (0–10 NRS). This change was decided with the hope that this modified response scale would allow more subtle changes to be captured by the questionnaire. The version of the SCQ that underwent cultural adaptation was a 32-item version, which was also the one that underwent psychometric validation.

The final version of the SCQ (after psychometric validation) is composed of 30 items covering 9 domains including: humanistic impact, exhaustion with caregiving role, lack of support, patient dependence, worries for the patient, perception of the care provided, financial dependence of the patient, financial impact of caregiving, overall difficulty of caregiving role.

In addition, a series of Caregiver Global Impression (CaGI) scales were also developed. These were designed for completion by caregivers to assess their perception of the severity of the schizophrenia symptoms of the person they care for over the past four weeks [“Please rate the severity of his/her symptoms during the past 4 weeks” scored “no symptoms” (0) to “very severe symptoms” (5)]; change in the schizophrenia symptoms of the person they care for since the beginning of the study [“Overall, how have his/her symptoms changed (if at all) since the beginning of the study (before starting treatment)?” scored from “very much improved” (1) to “very much worse” (7)]; and change in the experience of caring since the beginning of the study [“Overall, how much have your experiences of caring for a person with schizophrenia changed (if at all) since the beginning of the study (before the person started treatment)?” scored from “very much improved” (1) to “very much worse” (7)].

As for other conditions, clinical research in schizophrenia has become global [12]. In this context, outcome assessment has to be done in a multi-cultural framework; questionnaires need to be available in various languages to be included in multinational clinical trials and they need to appropriately capture the experience of individuals (patients or caregivers) with different linguistic and cultural backgrounds. Guidance for developing translations which are linguistically and culturally sound and respect the content validity of the original version has been developed by several organizations [13, 14]. In parallel, the Food and Drug Administration (FDA) requires that evidence about similarity of content validity and other measurement properties between the translated questionnaires and the original version be provided [10].

The objectives of the study were to prepare the SCQ and CaGI for use in a multicultural clinical research setting by conducting a proper translation of these instruments originally developed in US English in 11 languages, to present evidence that the translations capture the concepts of the original questionnaires and are well understood by caregivers of patients with schizophrenia in each target country. The translation of the SCQ was carried out on the instrument prior its psychometric validation (i.e., 32-item version).

## Methods

### Linguistic validation process

The instruments (SCQ and CaGI) were translated into languages that can be grouped into two “language families” (Indo-European and Uralic) based on their origins (Table 1).

**Table 1:** Language families and branches of the 11 target languages into which the SCQ and the CaGI scales were translated

Language family	Branch		Language (country)
Indo-European	Germanic	West	Dutch [DUT] (The Netherlands), English [ENG] (Australia, Canada, UK) German [GER] (Germany)
		North	Swedish [SWE] (Sweden)
	Italic	Romance	French [FRE] (Canada, France), Italian [ITA] (Italy), Spanish [SPA] (Spain)
Ural-Altaic	Uralic	Finnic	Finnish [FIN] (Finland)

**Fig. 1:**
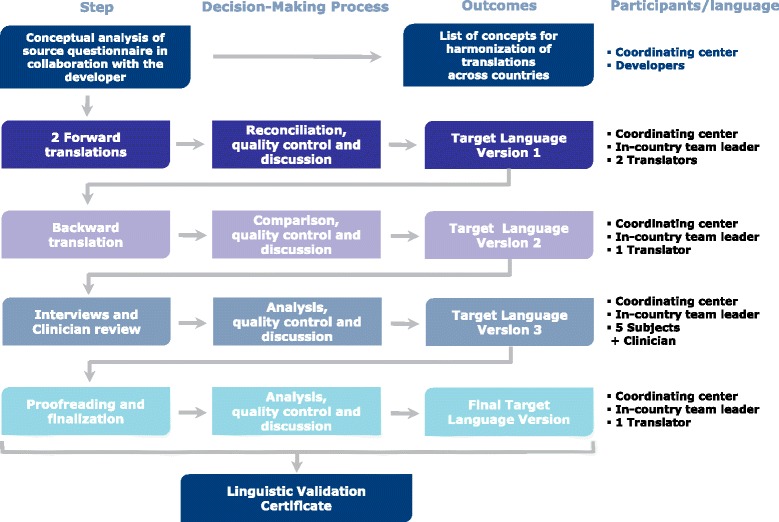
Standard linguistic validation process

The different language versions were developed using a standard or adjusted linguistic validation process fully complying with the International Society for Pharmacoeconomics and Outcomes Research (ISPOR) recommended procedures [13]. Fig. 1 illustrates the standard process. The conceptual analysis of the original questionnaires was performed with the developers of the original questionnaires in order to provide all translation teams with a document explaining the meaning of each item and suggesting terms to denote each concept. This was the basis for ensuring that all translations are harmonized between each other and faithful to the meaning of the original. Translations were developed by linguists through a process of dual forward translation (as per the ISPOR guidelines) and single back translation and reviews by a local team leader (linguist, psychologist or expert in survey research) and the project manager of the coordinating center who supervised all translations, in accordance with standards presented in Appendix A. The translations were then reviewed by a local clinician and tested via either telephone [French for Canada (n = 5); English for Canada (n = 5), Finnish (n = 2); Dutch (n = 1) and English for Australia (n = 1)] or face-to-face interviews of five caregivers of patients with schizophrenia living in the target countries. The convenience sample of 55 caregivers was recruited through the network of clinicians involved in the study. The interviews were conducted by the local team leader.

Language versions requiring the standard process were: French (Canada, France), German (Germany), Italian (Italy), Spanish (Spain), Dutch (the Netherlands), Finnish (Finland), and Swedish (Sweden).

For the adjusted process, the forward/backward step was replaced by an adaptation or localization step where an existing language version served as a basis for development of a separate local language version, e.g., US English version used as a basis to develop a UK English version. Language versions requiring an adjusted process were: English for Canada, UK, and Australia.

### Participants

Participants of the interviews had to be native-speaking residents of the target countries and informal caregivers of patients with schizophrenia. Subject selection criteria for a sample of five participants per language were equal representation of gender, and representing people of mixed education (i.e., a minimum of two participants with less than 15 years of education).

In each country, caregivers were asked to complete the local language version of the SCQ and the CaGI, to indicate if they had any difficulties in understanding the instructions, the items, and response scales, and to paraphrase each sentence in the questionnaires or reformulate it with their own words. In each country, the interviewer carefully explored if the subjects had a clear understanding of the concept behind each item (Were they able to provide the meaning of each item?), and if not why (i.e., inaccurate translation or concept not culturally relevant?). The interviewer also checked the cultural relevance of the translation and asked for alternative wording if needed. For each instruction, item and response category, all difficulties and suggestions for changes, were gathered in a summary grid and analyzed to produce the final versions.

### Analysis

All issues encountered for each questionnaire (SCQ, CaGI scales) during the full process and decisions made to solve them were summarized and were categorized as follows: Cultural (C), Idiomatic/Pragmatics (I), Semantic (S) and Syntactic (Sy). Table 2 illustrates each category.

**Table 2:** Categorization of translation difficulties

Category	Definition
**Cultural**	A word or formulation in the original is culturally loaded in the target context due to societal or religious taboos (i.e., eating habits in Asian countries, taboos in Muslim areas). The usage of certain words or phrases based on the culture of a given society may be improper in the target language.
	E.g.*, starchy foods (*e.g. *potato, bread, etc* *.), starchy foods (*e.g. *rice, pasta, chapatti, etc* *.).*
**Semantic**	Semantics concerns meanings, which are both denotative, i.e. the literal word (lexis), and connotative, namely the set of cultural and/or subjective associations implied by a word in addition to its literal explicit meaning. This category includes lexical differences and phraseology.
	E.g.*, meet your* * responsibilities* *, meet your* * duties* *, meet your* * obligations* *.*
**Idiomatic/Pragmatics**	The practicalities of how a language is used in its everyday context are different between the source and target language. For example, one language may have more social registers than another (there are a number of different forms of addressing a person in Japanese, whereas English may only have one) and the idiosyncrasies of one language (repetitions, focus on particular words, *etc.*) may not be found in another.
	E.g.*,* *I feel* * downhearted* and * blue* *,* * I feel* *down* and * sad.*
**Syntactic**	Correspond to specific aspects related to word morphology, sentence structure, grammar, punctuation. The structure and grammar of the source and target language diverge. For example, there is no grammatical form for the past tense in Tagalog.
	E.g.*, How flexible* * have you been finding* *…? How flexible* * have you found* *…?*

## Results

### Participants

Interviews were conducted with 55 caregivers of patients with schizophrenia from 10 countries representing 11 different languages (see Table 1). The participants ranged in age from 28 to 84 years and had 5 to 16 years of education (Table 3). Caregivers were predominantly female (69 %), which is consistent with previous research in samples of caregivers of patients with schizophrenia [15–17].

**Table 3:** Demographic characteristics of the caregivers* by target country

Country (language)	Age (years)	Gender (Males/Females)	Education (years)
Australia (English)	45 - 68	1/4	10 - 12
Canada (English)	28 - 52	2/3	11 - 14
Canada (French)	37 - 47	2/3	13 - 16
Finland (Finnish)	35 - 66	2/3	12
France (French)	54 - 71	2/3	9 - 12
Germany (German)	37 - 80	1/4	10 - 13
Italy (Italian)	35 - 67	2/3	5 - 13
The Netherlands (Dutch)	31 - 55	1/4	11 - 14
Spain (Spanish)	39 - 61	0/5	6 - 10
Sweden (Swedish)	40 - 60	2/3	9
UK (English)	64 - 84	2/3	9 - 14

*n = 5 for each language

### SCQ

Table 4 provides a summary of all difficulties that emerged during the translation process, in particular at the forward/backward translation steps and during the interviews. Fourteen out of the 32 items generated difficulties that raised discussions between the translators, the local team leader, and the central project manager. When an agreement could not be reached, the developer was consulted and gave the final decision. The difficulties were mostly of semantic origin (13 items). For instance, the meaning of “episode” (item 27) was found unclear or too technical by the caregivers and had to be qualified (i.e., psychotic, schizophrenic) or replaced by another word (i.e., crisis, relapse) to improve its clarity. The literal translation of “frustrated” (item 5) was not always possible and was replaced by the closest equivalent in the same semantic field. The only idiomatic expression present in the original version (i.e., “emotional rollercoaster” in item 31) was deleted in the languages of romance origin since it was not possible to find an idiomatic equivalent and the literal translation sounded too awkward (e.g., “montagne russe émotionnelle” in French).

**Table 4:** Most problematic SCQ items classified by type of difficulty (Diff.) - Cultural (C), Idiomatic/Pragmatics (I), Semantic (S) and Syntactic (Sy)

Item	Diff.	Lang.* (country)	LV^§^ Step		Description	Solution
			F/B	IS		
15. Over the past four weeks, how often did you feel that you didn’t have enough money to care for him/her, in addition to the rest of your expenses?	C	ENG (Canada)		✓	All respondents were somewhat confused by this item, since in Canada patients with schizophrenia automatically qualify for government assistance and their medications are covered by social insurance.	Item reworded as follows: “Over the past four weeks, how often did you feel that, in addition to the rest of your expenses, you needed more money to care for him/her?”
31. Over the past four weeks, how often did you experience emotional highs and lows (“an emotional rollercoaster”) because of his/her schizophrenia?	I	ENG (Canada), FRE (France), FIN, ITA, SPA, SWE	✓	✓	“High and lows” was translated either by a corresponding idiomatic expression or by a non idiomatic equivalent.	ENG, FRE (France): “ups and down”, FIN, ITA, SPA: “mood swings”, SWE: “valleys and tops”
		FRE (Canada, France), GER, ITA, SPA	✓	✓	“Emotional rollercoaster” was perceived as too idiomatic by translators and respondents of romance languages.	FRE (Canada), ITA, SPA: deleted, FRE (France): “up and down morale”, GER: “rollercoaster feelings”
1. Over the past four weeks, how often did you feel that he/she asked for more help than needed?	S	ITA, SPA	✓		Meaning of “he/she” was found unclear by translators.	“He/she” translated by “ill person” (Italian) [changed made for all items including he/she], “the person you take care of” (Spanish)
3. Over the past four weeks, how difficult was it for you to care for him/her and meet your other responsibilities?	S	ITA		✓	Meaning of “you” was considered too ambiguous by respondents.	“You” was removed, “him/her” replaced by “ill person”. Change made for all items with him/her.
4. Over the past four weeks, how embarrassed did you feel about his/her behavior?	S	DUT		✓	“Embarrassed” was translated with a word meaning both “ashamed” and “embarrassed”. The interviews suggested that the respondents gave it a meaning that had a strong connotation.	Replaced by a more direct equivalent of “embarrassed”
		GER		✓	Two respondents were disturbed by the translation of embarrassed (“ashamed”).	No change
5. Over the past four weeks, how frustrated did you feel about his/her behavior?	S	DUT		✓	The literal translation of frustrated was not understood by some respondents.	Translated by an equivalent of “dissatisfied”
		FRE (France)	✓	✓	The literal translation of frustrated was not possible. It was translated by an equivalent of “irritated”. The interviews suggested that the respondents gave it a meaning that had a strong connotation.	“Irritated” replaced by an equivalent of “annoyed”
11. Over the past four weeks, how often did you feel that you didn’t have as much privacy as you would have liked because of him/her?	S	DUT		✓	Respondents argued that the Dutch word did not convey the meaning of “time for oneself” and suggested another expression.	“Private life”
		FRE (France)	✓		The word “privacy” in French could also mean “intimacy”.	No change
14. Over the past four weeks, how often did you feel that he/she was overly dependent on you to help with daily activities?	S	DUT		✓	The meaning of “overly” was not clear for some respondents.	“Overly” replaced by an equivalent of “too”
20. Over the past four weeks, how often did you feel you should be doing more for him/her?	S	FRE (Canada)		✓	Item well understood. However the respondents found that its meaning was too close to the meaning of item 21.	“Doing more” was underlined in the translation
27. Over the past four weeks, how often did you worry that he/she might have an episode?	S	FRE (Canada)	✓		“Episode” was found unclear by translators.	Addition of “psychotic” to clarify meaning
		ENG (Canada), ITA, GER, SPA, SWE		✓	“Episode” was either perceived unclear or too technical by respondents.	ENG: “psychotic break”, “GER: “schizophrenic episode”, ITA SPA: “crisis”, SWE: “relapse”
7. Over the past four weeks, how often did you feel that his/her schizophrenia affected your relationship with other family members or friends in a negative way?	S	ENG (Canada, UK), FRE (Canada)		✓	Items well understood. However some respondents were strongly opposed to the use of “schizophrenia” because of the stigma attached to it.	No change [decision of the developer]
17. Over the past four weeks, how often did you feel you weren’t in control of your life because of his/her schizophrenia?	Sy	ITA	✓		The use of the courtesy form (3rd person singular) leads to an unwanted ambiguity: “your” is also translated as “his/her”, i.e., “your relationship” may be understood as “his/her relationship”, “his/her schizophrenia” as “your schizophrenia”.	“His/her” was deleted in the Italian version
28. Over the past four weeks, how often did you worry that his/her schizophrenia might get worse?						
31. Over the past four weeks, how often did you experience emotional highs and lows (“an emotional rollercoaster”) because of his/her schizophrenia?						
8. Over the past four weeks, how afraid were you of what the future holds for him/her?	Sy	DUT		✓	The item was well understood but the respondents found its structure unnecessarily too complicated.	“How worried were you about his/her future”

* Lang.: Language; ^§^ LV: Linguistic Validation; F/B: issues discussed at forward/backward steps; IS: issues discussed during interviews step

The various types of changes that were required are summarized in Table 4.

The clinician review did not reveal any problematic issue before the test with the caregivers.

An overview of the comprehension rate, language by language and item by item, is provided in Table 5. The SCQ was globally well-understood by most of the respondents (overall = 98.86 %).

**Table 5:** SCQ comprehension rates by languages and by items during interviews*

Items	DUT	ENG (Au)	ENG (Can)	ENG (UK)	FRE (Can)	FRE (Fran)	FIN	GER	ITA	SPA	SWE	Total by Item	
**1**	*4/5*	5/5	5/5	5/5	5/5	5/5	5/5	5/5	5/5	5/5	5/5	**54/55**	**98.18 %**
**2**	5/5	5/5	5/5	5/5	5/5	5/5	5/5	5/5	5/5	5/5	5/5	**55/55**	**100 %**
**3**	5/5	5/5	5/5	5/5	5/5	5/5	5/5	5/5	*3/5*	5/5	5/5	**53/55**	**96.36 %**
**4**	5/5	5/5	5/5	5/5	5/5	5/5	5/5	*3/5*	5/5	5/5	5/5	**53/55**	**96.36 %**
**5**	5/5	5/5	5/5	5/5	5/5	5/5	5/5	5/5	5/5	5/5	5/5	**55/55**	**100 %**
**6**	*3/5*	5/5	5/5	5/5	5/5	5/5	5/5	5/5	5/5	5/5	5/5	**53/55**	**96.36 %**
**7**	*4/5*	5/5	5/5	5/5	5/5	5/5	5/5	5/5	5/5	5/5	5/5	**54/55**	**98.18 %**
**8**	5/5	5/5	5/5	5/5	5/5	5/5	5/5	5/5	5/5	5/5	5/5	**55/55**	**100 %**
**9**	5/5	5/5	5/5	5/5	5/5	5/5	5/5	5/5	5/5	5/5	5/5	**55/55**	**100 %**
**10**	5/5	5/5	5/5	5/5	5/5	5/5	5/5	5/5	*4/5*	5/5	5/5	**54/55**	**98.18 %**
**11**	5/5	*4/5*	5/5	5/5	5/5	5/5	5/5	5/5	5/5	5/5	5/5	**54/55**	**98.18 %**
**12**	5/5	5/5	5/5	5/5	5/5	5/5	5/5	5/5	5/5	5/5	5/5	**55/55**	**100 %**
**13**	5/5	5/5	5/5	5/5	5/5	5/5	5/5	5/5	5/5	5/5	5/5	**55/55**	**100 %**
**14**	*3/5*	5/5	5/5	5/5	5/5	5/5	5/5	5/5	5/5	5/5	5/5	**53/55**	**96.36 %**
**15**	5/5	5/5	5/5	5/5	5/5	5/5	5/5	5/5	5/5	5/5	5/5	**55/55**	**100 %**
**16**	5/5	5/5	5/5	5/5	5/5	5/5	5/5	5/5	*4/5*	5/5	5/5	**54/55**	**98.18 %**
**17**	5/5	5/5	5/5	5/5	5/5	5/5	5/5	5/5	5/5	5/5	5/5	**55/55**	**100 %**
**18**	5/5	5/5	5/5	5/5	5/5	5/5	5/5	5/5	5/5	5/5	5/5	**55/55**	**100 %**
**19**	5/5	*4/5*	5/5	5/5	5/5	5/5	5/5	5/5	5/5	5/5	5/5	**54/55**	**98.18 %**
**20**	5/5	5/5	5/5	5/5	5/5	5/5	5/5	5/5	5/5	5/5	5/5	**55/55**	**100 %**
**21**	5/5	5/5	5/5	5/5	5/5	5/5	5/5	5/5	5/5	5/5	5/5	**55/55**	**100 %**
**22**	5/5	5/5	*4/5*	5/5	5/5	5/5	5/5	5/5	5/5	5/5	5/5	**54/55**	**98.18 %**
**23**	5/5	5/5	5/5	5/5	5/5	5/5	5/5	5/5	5/5	5/5	5/5	**55/55**	**100 %**
**24**	5/5	5/5	5/5	5/5	5/5	5/5	5/5	5/5	5/5	5/5	5/5	**55/55**	**100 %**
**25**	5/5	5/5	5/5	5/5	5/5	5/5	5/5	5/5	5/5	5/5	5/5	**55/55**	**100 %**
**26**	5/5	5/5	5/5	5/5	5/5	5/5	5/5	5/5	5/5	5/5	5/5	**55/55**	**100 %**
**27**	5/5	5/5	5/5	5/5	5/5	5/5	5/5	5/5	5/5	5/5	5/5	**55/55**	**100 %**
**28**	5/5	5/5	5/5	5/5	5/5	5/5	5/5	5/5	5/5	5/5	5/5	**55/55**	**100 %**
**29**	5/5	5/5	*4/5*	5/5	5/5	5/5	5/5	5/5	5/5	5/5	5/5	**54/55**	**98.18 %**
**30**	5/5	5/5	5/5	5/5	5/5	5/5	5/5	5/5	5/5	5/5	5/5	**55/55**	**100 %**
**31**	5/5	5/5	5/5	5/5	5/5	5/5	5/5	5/5	5/5	5/5	*2/5*	**52/55**	**94.54 %**
**32**	5/5	*4/5*	5/5	5/5	5/5	5/5	5/5	5/5	5/5	5/5	5/5	**54/55**	**98.18 %**
**Total by language**	**154/160**	**157/160**	**158/160**	**160/160**	**160/160**	**160/160**	**160/160**	**158/160**	**156/160**	**160/160**	**157/160**	**1740/1760**	**98.86 %**
	**96.25 %**	**98.13 %**	**98.75 %**	**100 %**	**100 %**	**100 %**	**100 %**	**98.75 %**	**97.50 %**	**100 %**	**98.13 %**	**98.86 %**	

*5 caregivers in each country

On the conceptual level, Anglophone caregivers in Canada argued about the relevance of item 26 (Over the past four weeks, how difficult was it to get him/her to take his/her medication?). They did not see the point of asking this question *(“They assume it’s a difficulty? That doesn’t make sense. Not sure what this question is getting at”*). Other caregivers in Finland and the Netherlands pointed out that item 26 was not relevant in case of injections. Interestingly, this item was deleted following the outcomes of the psychometric validation.

### CaGI scales

The translation of the CaGI scales in the 11 languages did not raise any major difficulty. Only five out of the 55 caregivers [one in Finland, four in Canada (two French, two English)] had difficulty understanding the meaning of the translations of “degree” in the expressions “degree of change in experience of caring” and “degree of change in symptoms”. Issues were resolved by either deleting “degree of” [English (Canada), Finnish], or using the word “evolution of” to replace “degree of change” [French (Canada)]. The term “evolution” was chosen for its neutral connotation implying either a worsening or an improvement. The overall comprehension rate of the CaGI scales was 99.17 %.

## Discussion

The results of the linguistic validation process indicate that the translations of the SCQ and the CaGI scales into 11 European languages adequately captured the concepts of the original English-language version of the questionnaires and were easily understood by caregivers of patients with schizophrenia. For each translation, it was important to utilize everyday language, yet remain true to the original meaning of the items. This involved focusing on semantics and required extensive discussion about the meaning of each concept and the adequate word to be chosen to convey it. This was accomplished through steady and continual discussion between the local team of translators, the local team leader, the central project manager and the developers of the original questionnaires. The initial conceptual analysis was crucial to obtaining translations harmonized with each other and faithful to the meaning of the original. The participation of caregivers of patients with schizophrenia greatly improved the initial translations, and provided input essential to the development of versions easily understood by the target population. In most cases, the solutions to each difficulty were decided collegially. Each decision was documented to enable further discussion at the time of the psychometric testing of the translations. The involvement of the caregivers in the development of the original version of the SCQ and its translations was essential. This is in the line with recent reviews which stress the need for tools with relevant content based on caregivers’ views [18, 19].

Cross-cultural equivalence is a critical factor/an essential component for instruments used in multi-cultural studies. Demonstrating cross-cultural equivalence entails investigating that the instrument measures the same concepts in a similar way across the different languages and cultures [20, 21]. It facilitates the pooling of data collected by the different languages versions of the instrument, and in the context of clinical trials, it optimizes the chance of demonstrating treatment benefit by improving the quality of measurement of the outcomes of interest [22]. Applying proper linguistic validation methodology contributes to the achievement of cross-culturally equivalent versions of instruments by optimizing the different languages versions. In addition, this process can generate useful data to document cross-cultural equivalence. During the linguistic validation of the SCQ and CaGI scales in 11 languages, no major cultural issues emerged. This shows that the concepts that were identified in the development of the original US English version of the questionnaire were relevant to the caregivers in other countries, supporting the existence of an overall common experience of the caregivers of patients with schizophrenia in a range of countries. This finding is important, given the recognized importance of cultural aspects in schizophrenia [23].

We have identified several limitations to our research: first, the use of a convenience sample (not representative of the entire population of caregivers of patients with schizophrenia), and the use of telephone interviews for some languages [French for Canada (n = 5); English for Canada (n = 5), Finnish (n = 2); Dutch (n = 1) and English for Australia (n = 1)], which may have prevented the interviewer to capture all the subtleties of body language during the completion of the questionnaire, and finally the fact that this research was conducted in Western countries only. It would be of great interest to extend this cross-cultural research on the experience of caregivers of patients with schizophrenia in a wider spectrum of countries and cultures. A recent review has shown that sociocultural and ethnic characteristics play an important role in the perception of family caregivers’ burden [24]. However, published research on cross-cultural perspectives in caring for someone with schizophrenia is still limited. A PubMed search with key words such as “schizophrenia,” “cross-cultural” and “caregiver” showed that publications in this area represent only 4 % (n = 38) of the papers published in schizophrenia and cross-cultural research (n = 942). Most of the studies operate within one continent [25–27] or within relatively similar cultural settings (i.e., Chinese-speaking countries) [28]. Very few compare populations such as ethnic groups within one country [29, 30] or caregivers from very different geographical areas or levels of development [31]. In a study comparing the health-related quality of life of French and Chilean caregivers, Boyer et al. [31] use a generic questionnaire (i.e., the SF-36) and show similar levels of health-related quality of life.

The availability in 11 European languages of disease-specific questionnaires such as the SCQ and the CaGI scales to assess the impact of caring for patients with schizophrenia is the first step to wider development and use in various cultural settings. International studies assessing differences of impact across cultures would be of great interest. They would enable cross-cultural comparisons, improve awareness, tracking, and management of impact on caregivers of patients with schizophrenia in different cultures, thus providing opportunity for increased support.

## Conclusions

Translations of the Schizophrenia Caregiver Questionnaire and the Caregiver Global Impression scales into 11 languages adequately captured the concepts in the original English version of the questionnaires, thereby demonstrating the conceptual, semantic, and cultural equivalence of each translation.

After psychometric testing, the instruments will be available for use in clinical trials facilitating international comparison and pooling of data and will provide new insights into the area of caregiving of schizophrenia.

## Appendix A. Translation procedures

1. Two translators who are native speakers of the target language and are experienced in translating health questionnaires independently translate the document.
2. After both translations are complete, the local team leader compares the two translations and produces a third translation. If difficulties arise, discussions with both translators take place to reach agreement. This process of discussion and review is known as “reconciling” and the resulting third translation is referred to as the target language version #1.
3. The translation is then given to a native English-speaking translator for translation back into English. This document is referred to as the “back-translation.”
4. The local team leader and the project manager of the coordinating center (central project manager) compare the original English to the back-translation and either approve or question each item in the back-translation.
5. The central project manager discusses concerns with the local (in-country) team leader who may change the translation, change the back-translation, or leave the translation as it is, providing a justification to the central project manager.
6. This process is repeated until all translation issues have been resolved and the revised back-translation is approved. The resulting translation is the target language version #2 which will be reviewed by a clinician and tested with subjects (interviews).

## Abbreviations

C: Cultural
CaGI: Caregiver Global Impression
Diff: Difficulty
F/B: Forward/backward step
FDA: Food and Drug Administration
I: Idiomatic/Pragmatics
IS: Interviews step
ISPOR: International Society for Pharmacoeconomics and Outcomes Research
Lang: Language
LV: Linguistic validation
SCQ: Schizophrenia Caregiver Questionnaire
S: Semantic
SF-36: Short Form (36) Health Survey
Sy: Syntactic
US: United States
UK: United Kingdom
ZBI: Zarit Burden Interview
